# A new scoring in differential diagnosis: multisystem inflammatory syndrome or adenovirus infection?

**DOI:** 10.55730/1300-0144.5905

**Published:** 2024-10-06

**Authors:** Mustafa GENÇELİ, Talha ÜSTÜNTAŞ, Özge METİN AKCAN, Sinan SAYLIK, Fatih ERCAN, Sevgi PEKCAN, Sipil GENÇELİ, Sevgi YAŞAR DURMUŞ, Mustafa ARGUN

**Affiliations:** 1 Department of Pediatric Infectious Diseases, Faculty of Medicine, Necmettin Erbakan University, Konya, Turkiye; 2 Department of Pediatrics, Faculty of Medicine, Necmettin Erbakan University, Konya, Turkiye; 3 Department of Pediatric Chest Diseases, Faculty of Medicine, Necmettin Erbakan University, Konya, Turkiye; 4 Department of Pediatrics, Konya City Hospital, Konya, Turkiye; 5 Department of Pediatrics Infectious Diseases, Kayseri City Hospital, Kayseri, Turkiye; 6 Department of Pediatrics Cardiology, Kayseri City Hospital, Kayseri, Turkiye

**Keywords:** Adenovirus infection, multisystem inflammatory syndrome in children, scoring

## Abstract

**Background/aim:**

Differentiating multisystem inflammatory syndrome in children (MIS-C) from adenovirus infection (AI) can be challenging due to similar clinical and laboratory findings. This study aimed to identify distinguishing characteristics and develop a scoring system to facilitate accurate diagnosis.

**Materials and methods:**

A comprehensive review of medical records was undertaken for 108 MIS-C patients and 259 patients with confirmed AI. A comparison of laboratory data and clinical findings was conducted across the patient groups using appropriate statistical tests.

**Results:**

The MIS-C patients were significantly older than the AI patients (p < 0.001). Diarrhea, rash, abdominal pain, vomiting, nonexudative conjunctivitis, lymphadenopathy tachycardia, bradycardia, hypotension, hypoxia seizures, agitation, headache, and altered consciousness symptoms were more frequently associated with MIS-C (p < 0.001), while cough and runny nose were significantly more common in AI (p < 0.001). Lymphopenia and thrombocytopenia were more prevalent in the MIS-C patients (p < 0.001). AI and MIS-C were scored by giving one point each to the parameters that created the difference. For AI, being ≤60 months of age, the presence of cough, runny nose and absence of diarrhea, rash, abdominal pain, vomiting, nonexudative conjunctivitis, lymphadenopathy, tachycardia, bradycardia, hypotension, hypoxia seizures, agitation, headache, and altered consciousness, lymphopenia, thrombocytopenia, and C-reactive protein value <124.5 mg/L were determined as each parameter plus one point. A total score above 14 could predict AI with a high degree of accuracy, with sensitivity at around 97.5% and specificity at 92.6%.

**Conclusion:**

The proposed inpatient scoring system, when used in conjunction with polymerase chain reaction testing, may improve the early differentiation of AI and MIS-C. This approach could help reduce unnecessary testing and optimize resource allocation. Further research with larger samples should investigate this novel scoring system to establish its reliability and generalizability.

## 1. Introduction

Adenovirus infection (AI) is a viral illness caused by a virus that commonly affects children. The disease often affects multiple body systems, causing symptoms such as upper respiratory tract infections, and gastrointestinal, genitourinary, neurologic, and ocular findings. The severity of AI can vary from mild to severe disease [1]. Acting as a proinflammatory virus, AI is known to trigger a cascade of defense mechanisms in children involving a surge of cytokines and chemokines, which leads to a heightened inflammatory state. In immunocompetent children, AI is usually self-limiting and resolves with supportive care [2]. However, AIs have been associated with strong inflammatory responses and morbidity in pediatric patients [3].

A rare but potentially life-threatening inflammatory condition, multisystem inflammatory syndrome in children (MIS-C) was identified during the COVID-19 pandemic. It is characterized by its potential to cause permanent organ damage or life-threatening illness. Patients with MIS-C often present with systemic involvement associated with a hyperinflammatory response and laboratory findings indicative of hyperinflammation. The diagnosis of MIS-C is based on a combination of clinical and laboratory findings [4,5].

Patients diagnosed with AI and MIS-C exhibit overlapping features, including high-grade fever, multiple lymphadenopathies, cutaneous and mucosal changes, conjunctivitis, and abdominal symptoms [6]. These overlapping features may lead to unnecessary hospitalizations and excessive testing, including blood tests, cardiac evaluations, and microbiologic examinations, increasing the burden on healthcare resources [4,6]. However, the severity of the disease usually differs between AI and MIS-C, with AI being milder and MIS-C being more severe. Importantly, they require different treatment approaches. AI typically requires only supportive care, and most patients can safely recover at home without the need for hospitalization, whereas MIS-C patients require hospitalization, close follow-up, intravenous immunoglobulin, immunomodulators, and antiplatelet agents [6].

This study focused on exploring certain clinical and laboratory features that differentiate AI from MIS-C in cases presenting with upper respiratory tract infections, aiming to develop a scoring system to aid in their differential diagnosis.

## 2. Materials and methods

Employing the Centers for Disease Control and Prevention’s diagnostic criteria, this retrospective cross-sectional study examined 108 patients with confirmed MIS-C [7] and 259 pediatric patients with polymerase chain reaction (PCR)-confirmed AI, detected in nasopharyngeal swab samples between April 2020 and July 2023. The study was conducted in two different tertiary hospitals that accept patients referred from other hospitals. Both had a pediatric intensive care unit, and pediatric cardiology and pediatric infection clinics.

All patients diagnosed with MIS-C were hospitalized regardless of their clinical classification. Patients diagnosed with MIS-C were classified as mild, moderate, and severe based on the clinical classification by Jonat et al. [8].

Data on the patients’ presenting complaints and laboratory parameters were collected. A scoring system was developed to predict AI based on the parameters that demonstrated significant differences across both patient groups.

Statistical analyses were performed using SPSS Statistics for Windows 18.0 (SPSS Inc., Chicago, IL, USA). Data normality was assessed through visual tools (histograms and probability plots) and common statistical analyses (Kolmogorov–Smirnov and Shapiro–Wilk tests). Nonnormally distributed numerical data were expressed as the median (1st–3rd quartile), while categorical data were expressed as frequencies and percentages. Nonnormally distributed numerical data were analyzed using the Mann–Whitney U test, while categorical data were compared using the chi-squared and Fisher’s exact tests. The diagnostic decision-making properties of C-reactive protein (CRP) as well as the total scores in predicting diseases were analyzed via receiver operating characteristic (ROC) curve analyses. Significant breakpoints were determined using the Youden index [max (sensitivity + specificity – 1)]. Sensitivity, specificity, and positive and negative predictive values of the determined breakpoints were calculated. Statistical significance was accepted as p < 0.05.

The study strictly adhered to the ethical principles outlined in the Declaration of Helsinki, and written approval was obtained from the local ethics committee prior to initiation of the research protocols (Decision No. 2023/4588).

## 3. Results

The sample consisted of 367 patients, comprising 108 (29.4%) with MIS-C and 259 (70.6%) with AI. Of the AI patients, 105 (40.5%) were hospitalized. Of the MIS-C patients, the clinical severity was mild in 38 (35.2%), moderate in 44 (40.8%) and severe in 26 (24%). AI and severe acute respiratory syndrome coronavirus 2 (SARS-CoV-2) PCR tests were applied to the MIS-C patients and all the results were negative. The MIS-C group had a significantly higher median age compared to the AI group (p < 0.001). The area under the ROC curve (AUC) value calculated for age in the MIS-C and AI groups was 0.858 (95% confidence interval (CI): 0.815–0.900; p < 0.001). Utilizing a cut-off value of 60 months for age yielded a sensitivity of 82.4% and a specificity of 73.4%. The sex distribution was similar in both groups (p = 0.145). The MIS-C group had a significantly higher incidence of diarrhea, rash, abdominal pain, vomiting, nonexudative conjunctivitis, lymphadenopathy, tachycardia, bradycardia, hypotension, hypoxia, seizures, agitation, headache, and altered of consciousness compared to the AI group (p < 0.001). In contrast, cough and runny nose were significantly more common symptoms in the AI group (p < 0.001). Fever and shortness of breath occurred at similar rates in both groups (p = 0.756 and p = 0.184, respectively). Comparison of the age, sex, and clinical characteristics of the MIS-C and AI groups is shown in Table 1.

The leukocyte count, neutrophil count, CRP, procalcitonin, ferritin and probrain natriuretic peptide (BNP) levels were significantly higher in the MIS-C group than in the AI group (p < 0.05). Leukopenia was present in 3.7% of the MIS-C patients and 2.9% of the AI patients, whereas neutropenia was present in 1.9% of the MIS-C patients and 3.8% of the AI patients. The rate of neutropenia and leukopenia were similar in both groups (p = 0.278 and p = 0.230, respectively). The incidence of lymphopenia and thrombocytopenia was higher in the MIS-C group than in the AI group (p < 0.001) (Table 2). The decision-making properties of CRP in predicting the diagnosis of MIS-C were analyzed via ROC analysis, which demonstrated the value of CRP in predicting MIS-C diagnosis, with an AUC of 0.908 (95% CI: 0.873–0.942, p < 0.001). A CRP cut-off value of 124.5 mg/L yielded sensitivity of 72.2%, specificity of 92.2%, a negative predictive value of around 87.1%, and positive predictive value of 82.1%.

Observed clinical and laboratory differences between the AI and MIS-C groups enabled the development of a new scoring system for differential diagnosis (Table 3). While there was a lack of sufficient ferritin, procalcitonin and pro-BNP data in the AI group for ROC analysis and inclusion, other parameters formed the basis of this scoring system. The distribution of patients whose ferritin, procalcitonin, and pro-BNP levels were measured is presented in Table 2.

The MIS-C group had a significantly lower median score of 11 (10–13) compared to the AI group, with a median score of 18 (17–19) (p < 0.001). ROC analysis demonstrated good diagnostic utility of the total score in predicting AI, with an AUC of 0.989 (95% CI: 0.978–0.999, p < 0.001). A total score above 14 yielded sensitivity of 97.5%, specificity of 92.6%, a positive predictive value of 96.1%, and negative predictive value of 95.2% for diagnosing AI.

## 4. Discussion

The identification of adenovirus by PCR in upper respiratory tract infections provides guidance for appropriate treatment, reduces hospital costs, and minimizes the overuse of antibiotics. However, PCR testing can be difficult to access and time-consuming. Although AI causes self-limiting infections in most patients, there are cases in which hyperinflammation is the main cause, requiring hospitalization. Systemic involvement and hyperinflammation may be confused with MIS-C, which requires different treatment approaches [3]. The development of clinical scores for patients with signs and symptoms mimicking Kawasaki disease, MIS-C, and AI is known to be a simple and cost-effective method to differentiate between patients who need close follow-up and more aggressive treatment and those who can be safely treated at home [5]. In the current study, it was aimed to develop a scoring system for the differentiation of these two conditions, which have similar aspects and can be difficult to distinguish during the pandemic period. This approach may prevent delays in MIS-C treatment, where early recognition and rapid management are critical, and ensure cost-effective management of AI, which usually requires only symptomatic treatment [3]. In the present case series, none of the patients presented with a concurrent diagnosis of AI and MIS-C. However, AI may develop during the course of MIS-C. The existing literature recommends that patients with overlapping clinical manifestations of AI and MIS-C be evaluated and managed as MIS-C [2]. Furthermore, echocardiographic assessment has been advocated for patients with fever persisting beyond 5 days, even in the presence of an AI diagnosis [6]. Similarly, while the etiology of Kawasaki disease remains elusive, the current recommendation is to treat it as such when AI is observed. The presence of AI should not deter clinicians from initiating necessary treatment when Kawasaki disease is strongly suspected [9]. A systematic review of the literature identified 3189 unique articles, of which 54 full-text articles were examined, and 18 observational studies were included. The weighted mean prevalence of viral positivity was 30% (95% CI: 14%–51%), ranging from 5% to 66%, with significant heterogeneity observed between studies. The highest individual virus positivity rates were observed for rhinovirus (19%), adenovirus (10%), and coronavirus (7%). The available data suggest that the patients in the current study also appeared to have received treatment similar to that for Kawasaki disease [10]. MIS-C is a hyperinflammatory state associated with COVID-19 infection. Its diagnosis involves a synthesis of clinical and laboratory parameters, often posing challenges [4,11]. Consequently, we propose that patients presenting with features suggestive of MIS-C, even in the context of AI, should be evaluated and managed accordingly.

Common clinical manifestations of MIS-C and AI include high-grade fever, multiple lymphadenopathies, cutaneous and mucosal changes, conjunctivitis, and abdominal symptoms. In a scoring system for outpatients with AI and patients with MIS-C and Kawasaki disease, the AI patients presented with runny nose (43%), vomiting (26.7%), diarrhea (26.7%), nonexudative conjunctivitis (20%), cervical lymphadenopathy (43.3%), and rash (10%). In contrast, the MIS-C patients presented with runny nose (0%), vomiting (50%), diarrhea (42.3%), nonexudative conjunctivitis (50%), cervical lymphadenopathy (23.1%), and rash (50%) [6]. Although these findings were similarly observed in both patient groups in the present study, diarrhea, rash, abdominal pain, vomiting, nonexudative conjunctivitis, lymphadenopathy, tachycardia, bradycardia, hypotension, hypoxia, seizures, agitation, headache, and altered consciousness were seen at significantly higher rates in the MIS-C patients, while cough and runny nose were observed at significantly higher rates in the AI patients.

In one study, patients with AI in the acute period had a leukocyte count of 12.880 mm^3^ (10.870–16.990), neutrophil count of 7.464 mm^3^ (5.522–9.406), lymphocyte count of 5.159 mm^3^ (3.916–6.402), CRP level of 8.3 mg/dL (5.9–10.6), and procalcitonin level of 1.2 ng/mL (0.3–3.15). The study emphasized that AI causes strong systemic inflammation in pediatric patients, with systemic inflammation and increased serum inflammatory markers observed even in mild AI [3]. AI causes a marked increase in the leukocyte count, and CRP and procalcitonin levels with neutrophilic dominance. Increased leukocyte count and neutrophilic dominance may be appropriate markers in AI-related upper respiratory tract infections in children [12,13]. Moreover, previous research reported a heightened inflammatory state involving a surge in the release of cytokines such as interleukin (IL)-6, IL-8, IL-10, and interferon gamma (IFN-γ) [3]. The majority of patients with MIS-C show evidence of inflammation on serum tests. Commonly elevated inflammatory markers in MIS-C include CRP, procalcitonin, the erythrocyte sedimentation rate, D-dimer, lactic acid dehydrogenase, and ferritin. Common hematologic findings include lymphopenia, leukocytosis, and thrombocytopenia [14,15]. In another study, laboratory findings of MIS-C were summarized as follows: elevated inflammatory markers (including IL-6, CRP, procalcitonin, erythrocyte sedimentation rate, ferritin, fibrinogen, and D-dimer), increased cardiac markers like troponin and pro-BNP (indicative of potential heart damage), and abnormal blood cell count (lymphopenia, neutrophilia, thrombocytopenia, mild anemia) [7,16]. Similarly, in the current study, leukocytosis with neutrophilic predominance and increased CRP levels were present in the AI group. Lymphopenia and thrombocytopenia were significantly more common in the MIS-C group than in the AI group. The MIS-C group also exhibited higher levels of inflammatory markers, including CRP, procalcitonin, ferritin, and pro-BNP.

A significant limitation of this study was the inability to establish cut-off values for ferritin, procalcitonin, and pro-BNP using the ROC analysis. It is hypothesized that MIS-C patients exhibit elevated levels of these markers due to severe hyperinflammation. However, it was not possible to confirm this, highlighting the need for more comprehensive investigations. While the number of COVID-19 cases has declined compared to the peak of the pandemic, they still persist, suggesting the potential for future MIS-C cases. This underscores the necessity for parameters that facilitate the differential diagnosis between MIS-C and AI. We anticipate that further research will refine this scoring system, contributing to easier and more accurate diagnosis of MIS-C.

Given the potential severity of MIS-C and the differences in treatment approaches between AI and MIS-C, it is crucial to quickly and accurately differentiate between these two conditions. The proposed scoring system, used in conjunction with PCR testing, has the potential to aid in early MIS-C identification and guide appropriate treatment. Further validation and replication of this scoring system in diverse patient populations is needed to confirm the scoring system’s broader clinical utility.

## Figures and Tables

**Table 1:** Comparison of age, sex, and clinical characteristics of the MIS-C and AI groups.

Variables		MIS-C* (n = 108)	AI ** (n = 259)	p
Age (months) (Median (IQR)		108.0 (72.0–143.7)	48 (24–72)	<0.001^a^
Sex, n (%)	Female	51 (47.2)	101 (39)	0.145^b^
	Male	57 (52.8)	158 (61)	
Fever, n (%)	No	1 (0.9)	3 (1.15)	0.756^b^
	Yes	107 (99.1)	256 (98.85)	
Cough, n (%)	No	84 (77.8)	71 (27.4)	<0.001^b^
	Yes	24 (22.2)	188 (72.6)	
Runny nose, n (%)	No	93 (86.1)	141 (54.4)	<0.001^b^
	Yes	15 (13.9)	118 (45.6)	
Shortness of breath, n (%)	No	88 (81.5)	225 (86.9)	0.184^b^
	Yes	20 (18.5)	34 (13.1)	
Diarrhea, n (%)	No	68 (63.0)	228 (88.0)	<0.001^b^
	Yes	40 (37.0)	31 (120)	
Rash, n (%)	No	32 (29.6)	247 (95.4)	<0.001^b^
	Yes	76 (70.4)	12 (4.6)	
Abdominal pain, n (%)	No	54 (50.0)	233 (90)	<0.001^b^
	Yes	54 (50.0)	26 (10)	
Vomiting, n (%)	No	48 (44.4)	241 (93.1)	<0.001^b^
	Yes	60 (55.6)	18 (6.9)	
Nonexudative conjunctivitis, n (%)	No	17 (15.79	237 (91.5)	<0.001^b^
	Yes	91 (84.3)	22 (8.5)	
Lymphadenopathy, n (%)	No	76 (70.3)	223 (86.1)	<0.001^b^
	Yes	32 (29.7)	36 (13.9)	
Headache, n (%)	No	87 (80.6)	253 (97.7)	<0.001^b^
	Yes	21 (19.4)	6 (2.3)	
Agitation, n (%)	No	91 (84.3)	253 (97.7)	<0.001^b^
	Yes	17 (15.7)	6 (2.3)	
Altered consciousness, n (%)	No	101 (93.5)	258 (99.6)	<0.001^c^
	Yes	7 (6.5)	1 (0.4)	
Seizure, n (%)	No	103 (95.4)	257 (99.2)	0.025^c^
	Yes	5 (4.6)	2 (0.8)	
Hypotension, n (%)	No	90 (83.3)	257 (99.2)	<0.001^b^
	Yes	18 (16.7)	2 (0.8)	
Bradycardia, n (%)	No	91 (84.3)	255 (98.5)	<0.001^b^
	Yes	17 (15.7)	4 (1.5)	
Tachycardia, n (%)	No	98 (90.7)	250 (96.5)	0.023^b^
	Yes	10 (9.3)	9 (3.5)	
Hypoxia, n (%)	No	92 (85.2)	250 (96.5)	<0.001^b^
	Yes	16 (14.8)	9 (3.5)	

a Mann–Whitney U test,

b chi-squared test,

c Fisher’s exact test,

* multisystem inflammatory syndrome in children (MIS-C),

** adenovirus infection (AI).

**Table 2:** Comparison of the laboratory parameters of MIS-C and AI groups.

Parameters	MIS-C (n = 108)	AI (n = 259)	p
	**Median (IQR)**	**Median (IQR)**	
Leukocyte count (mm)^3^	9480 (7097.5–12617.5)	10680 (7625–15275)	0.010^a^
Neutrophil count (mm)^3^	7405 (5470–10315)	5990 (3510–9710)	0.006^a^
Lymphocyte count (mm)^3^	875.0 (617.5–1475)	2830 (2110–4415)	<0.001^a^
Hemoglobin (g/dL)	11.9 (11–12.9)	11.9 (11–12.8)	0.949^a^
Platelets (mm)^3^	165.000 (122.000–224.000)	313.000 (250.000–417.000)	<0.001^a^
C-reactive protein (mg/L)	166.2 (108.8–226)	33 (9–59)	<0.001^a^
Procalcitonin (ug/L), n	4.02 (1.56–14), 103	0.29 (0.12–1.15), 29	<0.001^a^
Ferritin (ug/L), n	545 (267–901), 107	69.0 (42.5–153.5), 25	<0.001^a^
Pro-BNP (ng/L), n	3195.0 (1041–9126), 92	198 (94.0–312.7), 14	<0.001^a^
	**n (%)**	**n (%)**	
**Leukocyte**			
Leukopenia (<4000)	4 (3.7)	6 (2.9)	0.230^c^
Normal	55 (50.9)	87 (41.6)	
Leukocytosis (>10000)	49 (45.4)	116 (55.5)	
**Neutropenia (<1500)**			
No	106 (98.1)	201 (96.2)	0.278^b^
Yes	2 (1.9)	8 (3.8)	
**Lymphopenia (<1500)**			
No	25 (23.1)	187 (89.5)	<0.001^c^
Yes	83 (76.9)	22 (10.5)	
**Thrombocytopenia (<150000)**			
No	60 (55.6)	199 (95.2)	<0.001^c^
Yes	48 (44.4)	10 (4.8)	

a Mann–Whitney U test,

b Fisher’s exact test,

c chi-squared test.

**Table 3:** A scoring system to predict AI.

Age	≤60 months=1
Cough	Yes = 1
Runny nose	Yes = 1
Diarrhea	None = 1
Rash	None = 1
Abdominal pain	None = 1
Vomiting	None = 1
Nonexudative conjunctivitis	None = 1
Lymphadenopathy	None = 1
Headache	None = 1
Agitation	None = 1
Altered consciousness	None = 1
Seizure	None = 1
Hypotension	None = 1
Bradycardia	None = 1
Tachycardia	None = 1
Hypoxia	None = 1
Lymphopenia	None = 1
Thrombocytopenia	None = 1
C-reactive protein	<124.5 mg/L = 1
